# Supplementary figures and images for: Vegetation responses to season of fire in an aseasonal, fire-prone fynbos shrubland
Source: PeerJ. 2017 Aug 10;5:e3591. doi: 10.7717/peerj.3591 (PMC5554598; doi:10.7717/peerj.3591)

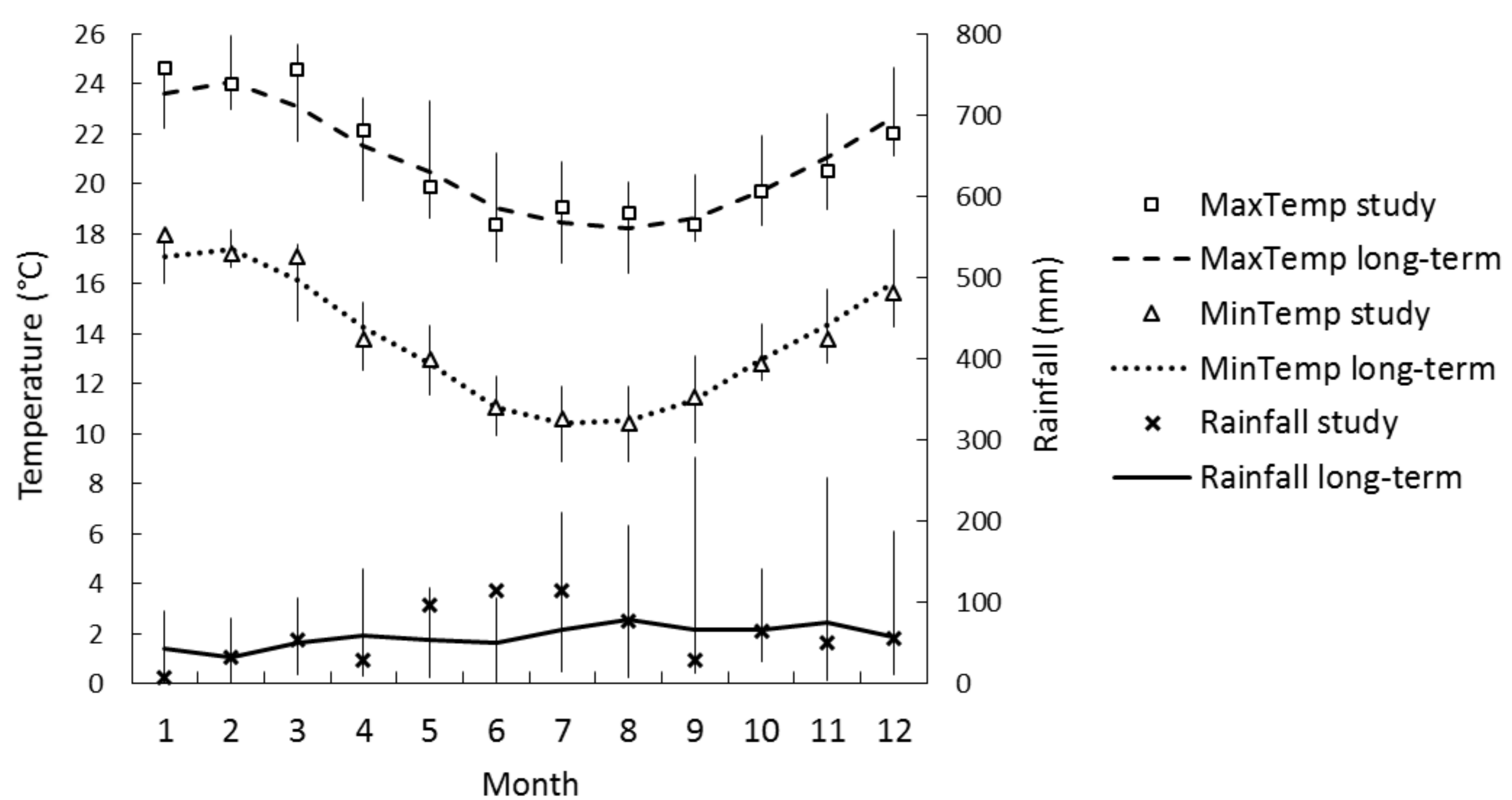

Supplement: Figure S1 — Mean monthly minimum and maximum temperatures and rainfall compared between the study period of the seed planting experiment and the remainder of the long-term record available for the nearest and most central weather station (Plettenberg Bay, 1993–2013). Bars show minimum and maximum recorded figures for long-term data. [file peerj-05-3591-s001.pdf]

Live recruits (% of planted seeds)

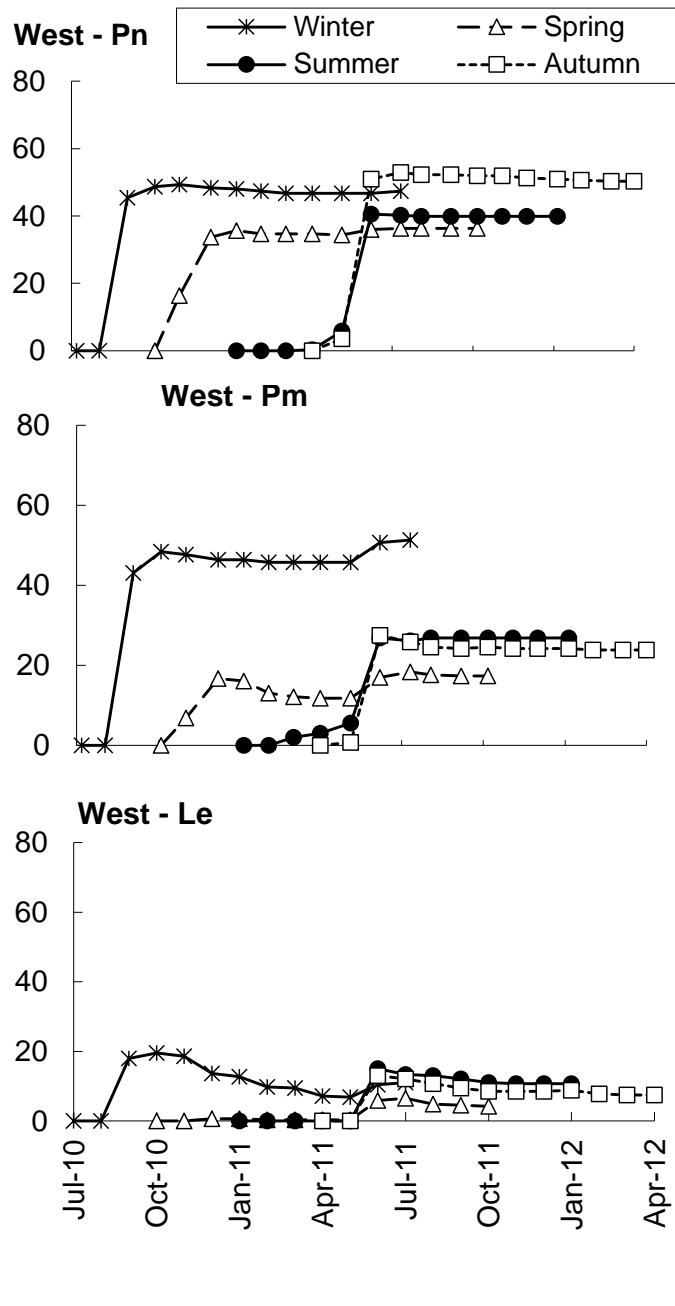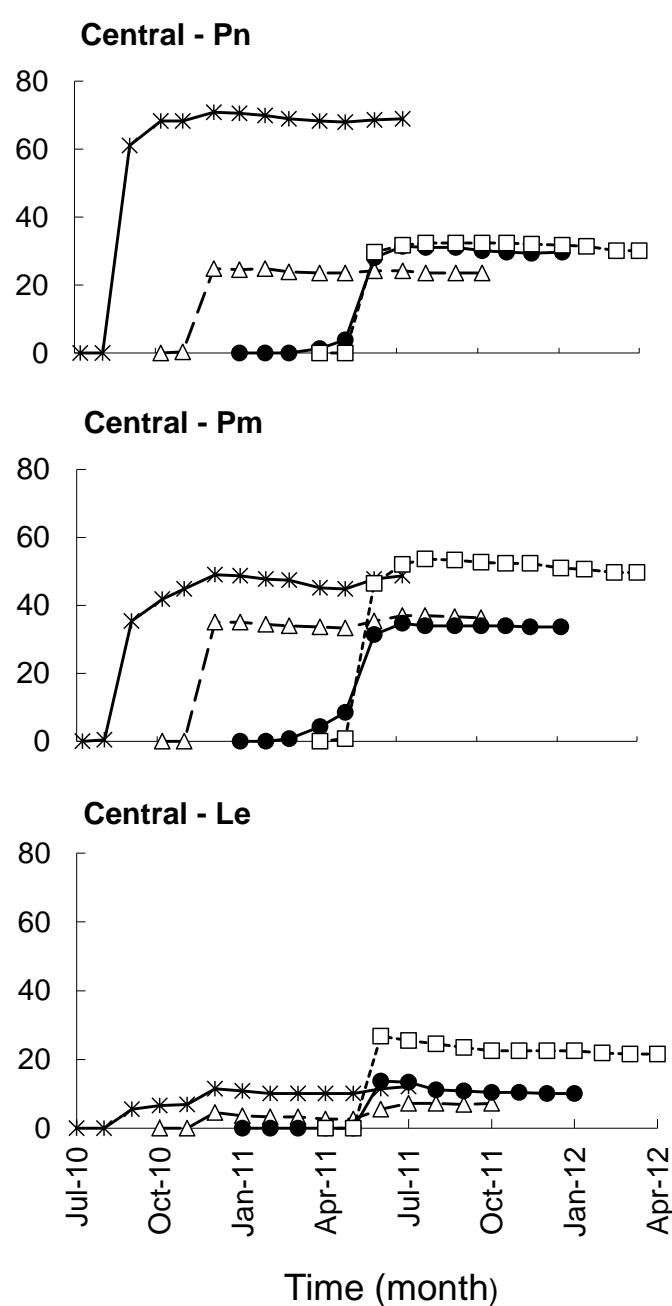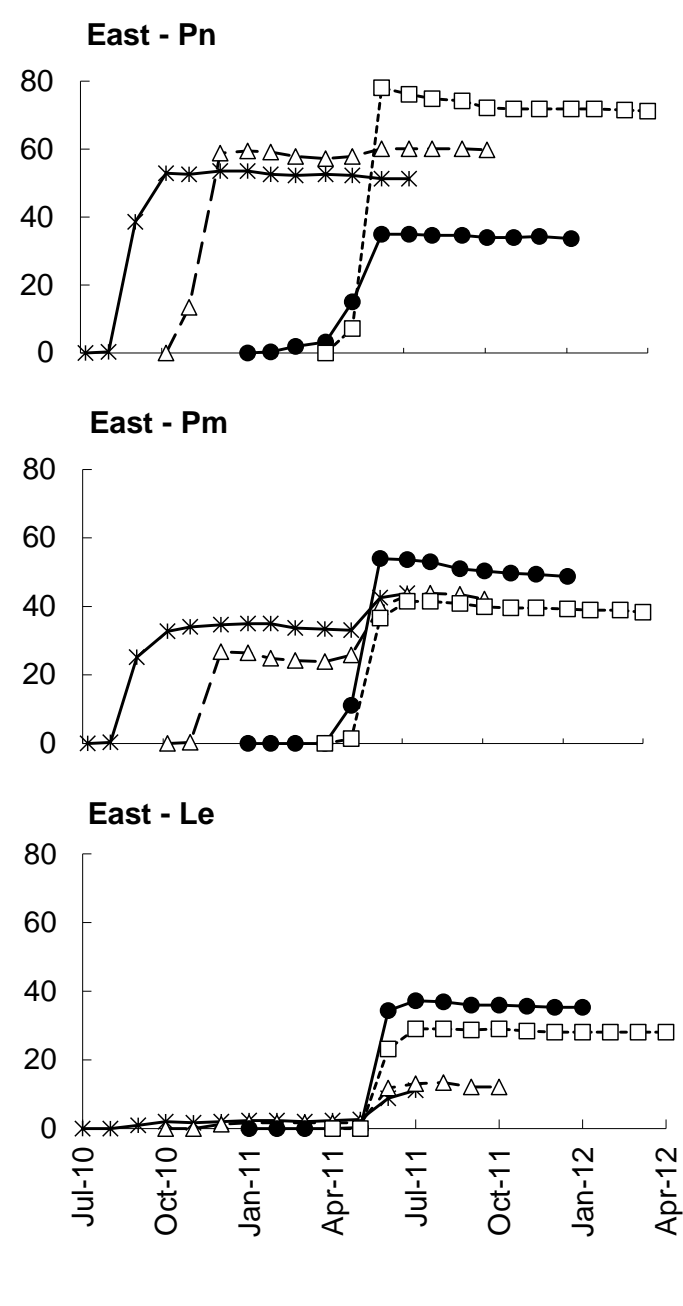

Time (month)

Supplement: Figure S2 — Live recruits of three Proteaceae species (Le, Leucadendron eucalyptifolium; Pm, P. mundii; Pn, Protea neriifolia) observed within the first week of each month, expressed as a percentage of seeds planted under predator-exclosures after clearing of above-ground vegetation (simulating fire) in four seasons at three sites (West, Central, East). Zero values at the start of each series mark planting occasions for each of the austral seasons. [file peerj-05-3591-s002.pdf]
