# Supplementary material for: Vegetation responses to season of fire in an aseasonal, fire-prone fynbos shrubland
Source: PeerJ. 2017 Aug 10;5:e3591. doi: 10.7717/peerj.3591 (PMC5554598; doi:10.7717/peerj.3591)
Supplement: Table S1 — The number of belt transects (each 2 m × 30 m), the season of fire, and the proteoid species (Le, Leucadendron eucalyptifolium; Lu, L. uliginosum; Pe, Protea eximia; Pm, P. mundii; Pn, P. neriifolia) surveyed at each site are shown . [file peerj-05-3591-s003.docx]

**Table S1. Locations of post-fire recruitment survey sites.** The number of belt transects (each 2 m x 30 m), the season of fire, and the proteoid species (Le, *Leucadendron eucalyptifolium*; Lu, *L. uliginosum*; Pe, *Protea eximia*; Pm, *P. mundii*; Pn, *P. neriifolia*) surveyed at each site are shown.

| Site | Latitude (°S) | Longitude (°E) | No. Transects | Season of Fire | Species | | | | |
| --- | --- | --- | --- | --- | --- | --- | --- | --- | --- |
|  |  |  |  |  | *Le* | *Lu* | *Pe* | *Pm* | *Pn* |
| 1 | 34.02114 | 23.91021 | 2 | Winter | x |  |  |  | x |
| 2 | 33.98578 | 23.04614 | 6 | Winter | x |  |  |  | x |
| 3 | 33.96381 | 24.26465 | 3 | Winter |  |  |  | x | x |
| 4 | 34.02801 | 24.44962 | 2 | Spring | x |  |  | x | x |
| 5 | 33.86331 | 22.81808 | 14 | Spring | x |  |  |  |  |
| 6 | 33.91472 | 23.71194 | 2 | Spring |  | x |  |  |  |
| 7 | 33.93611 | 23.55361 | 2 | Spring | x |  |  | x |  |
| 8 | 33.93389 | 23.54361 | 2 | Spring | x |  |  | x |  |
| 9 | 33.83654 | 22.72529 | 5 | Spring |  | x |  |  |  |
| 10 | 33.85940 | 22.61645 | 4 | Spring | x | x |  |  |  |
| 11 | 33.88000 | 22.71530 | 2 | Spring |  | x | x |  | x |
| 12 | 33.88000 | 22.71530 | 2 | Spring | x | x | x | x | x |
| 13 | 33.91702 | 23.13348 | 5 | Summer | x |  |  |  |  |
| 14 | 33.91537 | 23.13504 | 6 | Summer | x |  |  |  |  |
| 15 | 33.85194 | 22.91842 | 5 | Summer | x |  |  |  |  |
| 16 | 33.85272 | 22.92063 | 6 | Summer | x |  |  |  |  |
| 17 | 33.88000 | 22.71530 | 2 | Summer | x | x |  |  |  |
| 18 | 33.85430 | 22.94191 | 5 | Summer | x |  |  |  |  |
| 19 | 33.85645 | 22.63816 | 6 | Summer |  |  |  | x | x |
| 20 | 33.88000 | 22.71530 | 2 | Summer | x | x |  |  | x |
| 21 | 33.83716 | 22.72121 | 3 | Autumn |  | x |  |  |  |
| 22 | 33.83850 | 22.71530 | 3 | Autumn | x |  |  |  |  |
| 23 | 33.97027 | 23.21572 | 5 | Autumn | x |  |  | x |  |
| 24 | 33.96858 | 23.21896 | 3 | Autumn | x |  |  |  | x |
| 25 | 33.95803 | 24.06697 | 4 | Autumn | x |  |  |  |  |
| 26 | 33.96008 | 24.05852 | 3 | Autumn | x |  |  |  |  |
