# Supplementary material for: Vegetation responses to season of fire in an aseasonal, fire-prone fynbos shrubland
Source: PeerJ. 2017 Aug 10;5:e3591. doi: 10.7717/peerj.3591 (PMC5554598; doi:10.7717/peerj.3591)
Supplement: Table S2 — ∗ 0 outside the confidence interval ǂ Numbers in parentheses after the estimated regression coefficients (βs) are asymptotic 95% confidence intervals. Models fitted using treatment contrasts. The (Intercept) therefore represents the log-odds of recruitment for Species = Le during Season = Winter at Site = West. For the mixed model, Var in the lower part of the table gives the variance of intercepts in groups of records identified by the referenced group in the random-effects structure. That is, the entry Plot:Site (Intercept) refers to the variance in the 12 intercepts of Plot within each level of Site in the second-level grouping of the random-effects structure (1—Site/Plot/Season/PlotExcl). [file peerj-05-3591-s004.docx]

**Table S2.** Summaries of matching fixed-effects [Recruitment ~ Season × Species × Site] and mixed-effects [Recruitment ~ Season × Species × Site + (1|Site/Plot/Season/PlotExcl)] logistic regression models of the effects of planting season (winter, spring, summer, autumn), species (Le, *Leucadendron eucalyptifolium*; Lu, *L. uliginosum*; Pe, *Protea eximia*; Pm, *P. mundii*; Pn, *P. neriifolia*), and site (West, Central, East) on recruitment (survival at one year post-planting)^ǂ^.

|  | Fixed-effects | Mixed-effects |
| --- | --- | --- |
| (Intercept) | *−*2*.*11 [*−*2*.*47; *−*1*.*75]∗ | *−*2*.*14 [*−*2*.*56; *−*1*.*73]∗ |
| SeasonSpring | *−*1*.*09 [*−*1*.*77; *−*0*.*40]∗ | *−*1*.*09 [*−*1*.*83; *−*0*.*35]∗ |
| SeasonSummer | *−*0*.*00 [*−*0*.*51; 0*.*51] | *−*0*.*01 [*−*0*.*60; 0*.*57] |
| SeasonAutumn | *−*0*.*40 [*−*0*.*95; 0*.*16] | *−*0*.*39 [*−*1*.*02; 0*.*24] |
| Species Pm | 2*.*15 [1*.*73; 2*.*58]∗ | 2*.*18 [1*.*75; 2*.*61]∗ |
| SpeciesPn | 1*.*98 [1*.*56; 2*.*41]∗ | 2*.*01 [1*.*58; 2*.*44]∗ |
| SiteCentral | 0*.*13 [*−*0*.*37; 0*.*63] | 0*.*14 [*−*0*.*44; 0*.*71] |
| SiteEast | *−*0*.*00 [*−*0*.*51; 0*.*51] | *−*0*.*01 [*−*0*.*60; 0*.*58] |
| SeasonSpring:SpeciesPm | *−*0*.*52 [*−*1*.*29; 0*.*26] | *−*0*.*54 [*−*1*.*31; 0*.*24] |
| SeasonSummer:SpeciesPm | *−*1*.*04 [*−*1*.*66; *−*0*.*43]∗ | *−*1*.*06 [*−*1*.*67; *−*0*.*44]∗ |
| SeasonAutumn:SpeciesPm | *−*0*.*80 [*−*1*.*46; *−*0*.*15]∗ | *−*0*.*82 [*−*1*.*48; *−*0*.*16]∗ |
| SeasonSpring:SpeciesPn | 0*.*65 [*−*0*.*10; 1*.*41] | 0*.*65 [*−*0*.*10; 1*.*41] |
| SeasonSummer:SpeciesPn | *−*0*.*28 [*−*0*.*88; 0*.*32] | *−*0*.*27 [*−*0*.*88; 0*.*33] |
| SeasonAutumn:SpeciesPn | 0*.*54 [*−*0*.*10; 1*.*18] | 0*.*54 [*−*0*.*11; 1*.*18] |
| SeasonSpring:SiteCentral | 0*.*51 [*−*0*.*37; 1*.*39] | 0*.*50 [*−*0*.*47; 1*.*47] |
| SeasonSummer:SiteCentral | *−*0*.*20 [*−*0*.*92; 0*.*52] | *−*0*.*20 [*−*1*.*02; 0*.*63] |
| SeasonAutumn:SiteCentral | 1*.*09 [0*.*38; 1*.*80]∗ | 1*.*07 [0*.*25; 1*.*89]∗ |
| SeasonSpring:SiteEast | 1*.*21 [0*.*37; 2*.*06]∗ | 1*.*23 [0*.*29; 2*.*17]∗ |
| SeasonSummer:SiteEast | 1*.*51 [0*.*84; 2*.*17]∗ | 1*.*55 [0*.*76; 2*.*33]∗ |
| SeasonAutumn:SiteEast | 1*.*54 [0*.*83; 2*.*25]∗ | 1*.*55 [0*.*73; 2*.*37]∗ |
| SpeciesPm:SiteCentral | *−*0*.*22 [*−*0*.*81; 0*.*37] | *−*0*.*23 [*−*0*.*82; 0*.*37] |
| SpeciesPn:SiteCentral | 0*.*74 [0*.*14; 1*.*34]∗ | 0*.*75 [0*.*15; 1*.*35]∗ |
| SpeciesPm:SiteEast | *−*0*.*30 [*−*0*.*90; 0*.*30] | *−*0*.*30 [*−*0*.*91; 0*.*30] |
| SpeciesPn:SiteEast | 0*.*17 [*−*0*.*43; 0*.*77] | 0*.*18 [*−*0*.*42; 0*.*79] |
| SeasonSpring:SpeciesPm:SiteCentral | 0*.*58 [*−*0*.*43; 1*.*59] | 0*.*60 [*−*0*.*40; 1*.*61] |
| SeasonSummer:SpeciesPm:SiteCentral | 0*.*62 [*−*0*.*24; 1*.*48] | 0*.*63 [*−*0*.*24; 1*.*49] |
| SeasonAutumn:SpeciesPm:SiteCentral | 0*.*15 [*−*0*.*70; 1*.*00] | 0*.*19 [*−*0*.*67; 1*.*04] |
| SeasonSpring:SpeciesPn:SiteCentral | *−*2*.*01 [*−*3*.*01; *−*1*.*01]∗ | *−*2*.*03 [*−*3*.*03; *−*1*.*02]∗ |
| SeasonSummer:SpeciesPn:SiteCentral | *−*1*.*12 [*−*1*.*98; *−*0*.*26]∗ | *−*1*.*14 [*−*2*.*00; *−*0*.*28]∗ |
| SeasonAutumn:SpeciesPn:SiteCentral | *−*2*.*82 [*−*3*.*66; *−*1*.*97]∗ | *−*2*.*83 [*−*3*.*69; *−*1*.*98]∗ |
| SeasonSpring:SpeciesPm:SiteEast | 0*.*31 [*−*0*.*67; 1*.*28] | 0*.*31 [*−*0*.*67; 1*.*29] |
| SeasonSummer:SpeciesPm:SiteEast | *−*0*.*25 [*−*1*.*07; 0*.*56] | *−*0*.*26 [*−*1*.*08; 0*.*56] |
| SeasonAutumn:SpeciesPm:SiteEast | *−*0*.*55 [*−*1*.*41; 0*.*30] | *−*0*.*55 [*−*1*.*41; 0*.*30] |
| SeasonSpring:SpeciesPn:SiteEast | *−*0*.*48 [*−*1*.*44; 0*.*48] | *−*0*.*49 [*−*1*.*45; 0*.*48] |
| SeasonSummer:SpeciesPn:SiteEast | *−*1*.*96 [*−*2*.*77; *−*1*.*15]∗ | *−*2*.*00 [*−*2*.*82; *−*1*.*19]∗ |
| SeasonAutumn:SpeciesPn:SiteEast | *−*0*.*81 [*−*1*.*66; 0*.*03] | *−*0*.*80 [*−*1*.*66; 0*.*05] |
| AIC | 795.09 | 1802.27 |
| BIC | 891.65 | 1953.5 |
| Log Likelihood | -361.55 | -861.13 |
| Deviance | 218.3 |  |
| Num. obs. | 108 | 324 |
| Num. groups: PlotExcl:(Season:(Plot:Site)) |  | 108 |
| Num. groups: Season:(Plot:Site) |  | 36 |
| Num. groups: Plot:Site |  | 36 |
| Num. groups: Site |  | 3 |
| Var: PlotExcl:(Season:(Plot:Site)) (Intercept) |  | 0.08 |
| Var: Season:(Plot:Site) (Intercept) |  | 0 |
| Var: Plot:Site (Intercept) |  | 0.01 |
| Var: Site (Intercept) |  | 0 |

∗ 0 outside the confidence interval

^ǂ^ Numbers in parentheses after the estimated regression coefficients (βs) are asymptotic 95% confidence intervals. Models fitted using treatment contrasts. The (Intercept) therefore represents the log-odds of recruitment for Species = Le during Season = Winter at Site = West. For the mixed model, Var in the lower part of the table gives the variance of intercepts in groups of records identified by the referenced group in the random-effects structure. That is, the entry Plot:Site (Intercept) refers to the variance in the 12 intercepts of Plot within each level of Site in the second-level grouping of the random-effects structure (1|Site/Plot/Season/PlotExcl).
