# Supplementary material for: Vegetation responses to season of fire in an aseasonal, fire-prone fynbos shrubland
Source: PeerJ. 2017 Aug 10;5:e3591. doi: 10.7717/peerj.3591 (PMC5554598; doi:10.7717/peerj.3591)
Supplement: Table S3 [file peerj-05-3591-s005.docx]

**Table S3.** Output of generalized linear model (fitted by quasi-likelihood and the square link) of the effect of fire season on post-fire recruitment success (expressed as seedling-parent ratio).

|  | Estimate | SE | t | Pr(>\|t\|) |
| --- | --- | --- | --- | --- |
| (Intercept) | 3.00 | 1.38 | 2.18 | 0.0349 |
| Fire season Spring | 10.27 | 3.78 | 2.72 | 0.0094 |
| Fire season Summer | 3.74 | 2.45 | 1.53 | 0.1340 |
| Fire season Autumn | 8.20 | 4.66 | 1.76 | 0.0855 |
